# Supplementary material for: Gate-tunable anomalous Hall effect in Bernal tetralayer graphene
Source: Nat Commun. 2023 Dec 1;14:7925. doi: 10.1038/s41467-023-43796-w (PMC10692167; doi:10.1038/s41467-023-43796-w)
Supplement: Supplementary file 1 — Supplementary Information [file 41467_2023_43796_MOESM1_ESM.pdf]

Supplementary Information

Gate-tunable anomalous Hall effect in Bernal tetralayer graphene

Hao Chen [1,ζ], Arpit Arora [2,ζ], Justin C.W. Song [2,*] and Kian Ping Loh [1,*]

[1] Department of Chemistry, National University of Singapore, Singapore 117543.

[2] Division of Physics and Applied Physics, School of Physical and Mathematical Sciences, Nanyang Technological University, Singapore, Singapore 637371

[ζ] These authors contributed equally.

* These authors jointly supervised this work.

Correspondence to: justinsong@ntu.edu.sg; chmlohkp@nus.edu.sg

1. Device fabrication

The tetralayer graphene flakes are exfoliated on silicon dioxide substrate and examined under an optical microscope. To identify the layer thickness, both optical contrast and half peak width in 2D peak in Raman microscopy are employed. Then the hexagonal boron nitride flakes exfoliated on substrate are also examined with optical microscope to ensure high quality. Further, narrow few layer graphene strips (thicker than eight layers) are prepared as metallic top and bottom gates. Lastly the assembly process is done in dry transfer with a PC stamp. Note the top and bottom graphite is intentionally placed to overlap with the BTG sample. The whole stack is then annealed under ultrahigh vacuum, followed by etching and metal evaporation. Some optical graphs of fabricated devices are shown in Supplementary Fig. 1.

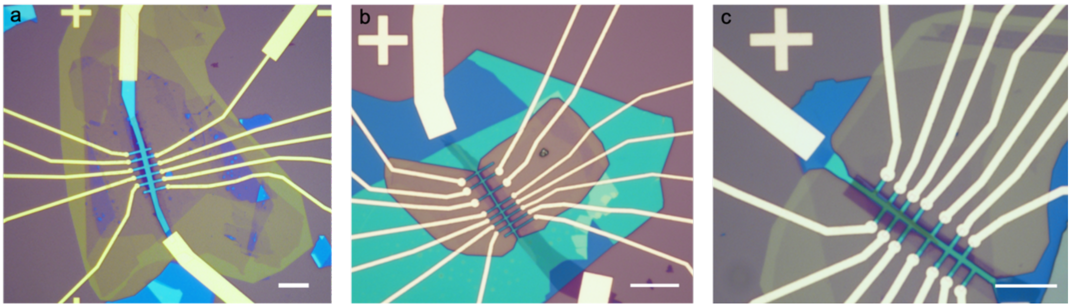

Supplementary Figure 1. Optical graphs of fabricated BTG devices. Scale bar is 10 μm.

2. Resistivity in (*n*, *D*) parameter space with filling fraction displayed

In Supplementary Fig. 2, the same resistivity plot as Figure 2a of the main text is displayed but with the filling fraction displayed on the top x-axis.

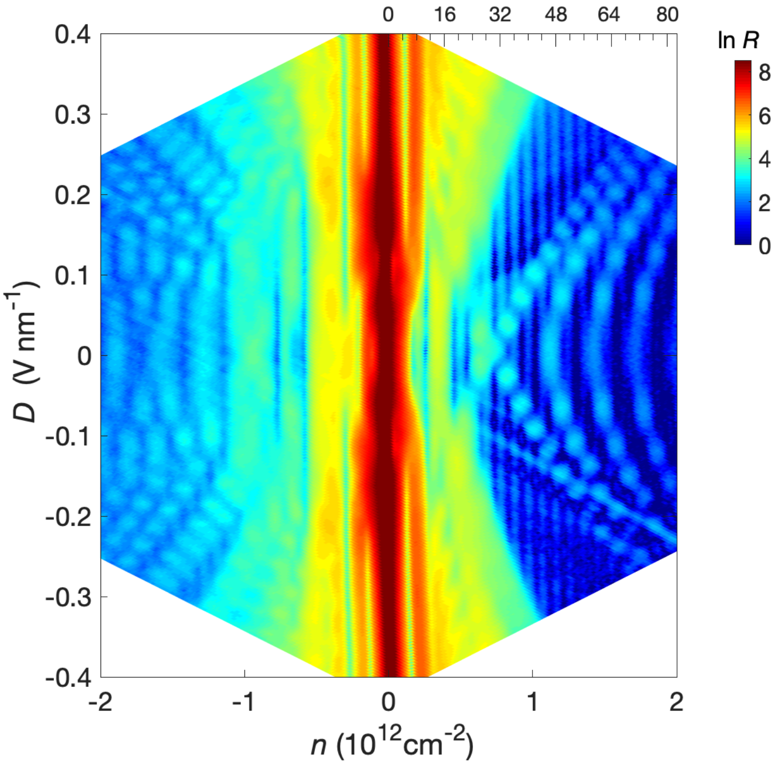

Supplementary Figure 2, Four-terminal resistance as function of *n* and *D* conditions at 0.3 K. Reproduced from Fig. 2a with filling factors denoted in the upper x-axis. The spacing between small ticks is 4.

3. Fast Fourier Transform (FFT) analysis

FFT analysis of Shubnikov de Haas oscillations are obtained in the following approach. First, the magnetoresistance data are obtained at fixed (*n*, D) conditions denoted in Supplementary Fig. 3a&b. Then a background is subtracted from the $R_{xx}(1/B)$ data following the method described in Ref. 1. The data is interpolated to produce an even grid as a function of $1/B$ (Supplementary Fig. 3c&d). Then the

extracted frequency (f_B) and magnitude from FFT is obtained and factorized to be $f = f_B / n\phi_0$, which is shown in Figure 2b in the main text.

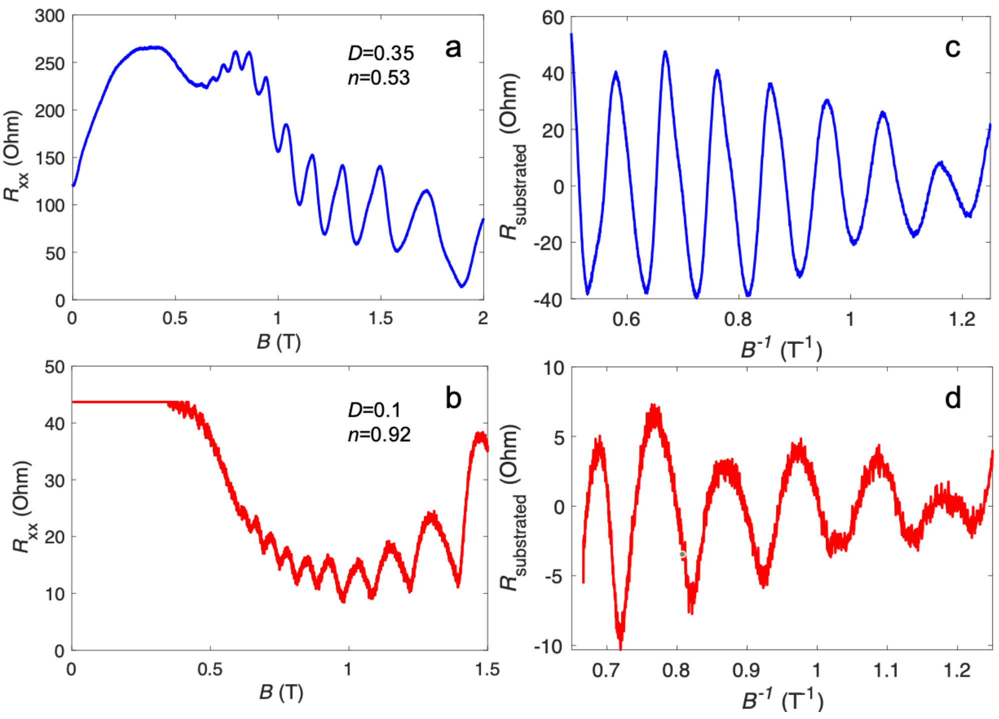

Supplementary Figure 3. Quantum oscillations data analysis. a&b, Resistance as a function of *B* at fixed *n* and *D* conditions corresponding to blue up-triangle (valley metal) and red dot (full metal) in Fig. 2 in main text, respectively. **b&c**, Extracted resistance after background subtraction for **a&b**, respectively.

4. Displacement field dependence of AHE

In addition to the carrier density dependence of AHE shown in Figure 3 in main text, we also investigate the *D*-field dependence of AHE. Hall resistance is measured at fixed density but for varying *D*-field as shown in Supplementary Fig. 4. Notably, AHE is switched on at finite *D*-field, becomes larger and can be seen to develop moderate staircase-like features with increasing *D*-field. In Supplementary Fig. 5, we show R_{AH} for both positive and negative *D*-field.

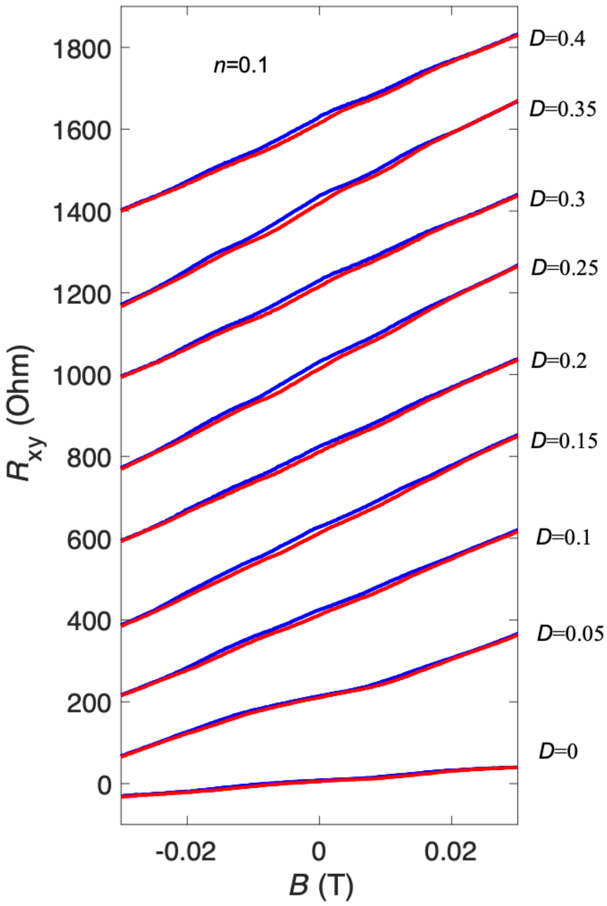

Supplementary Figure 4. *D*-field dependence of Hall resistance. Hall resistance (R_{xy}) as a function of *B* at fixed n=0.1×10^{12} cm^{-2} but different positive *D* field conditions. Each pair of curves are shifted vertically for better view.

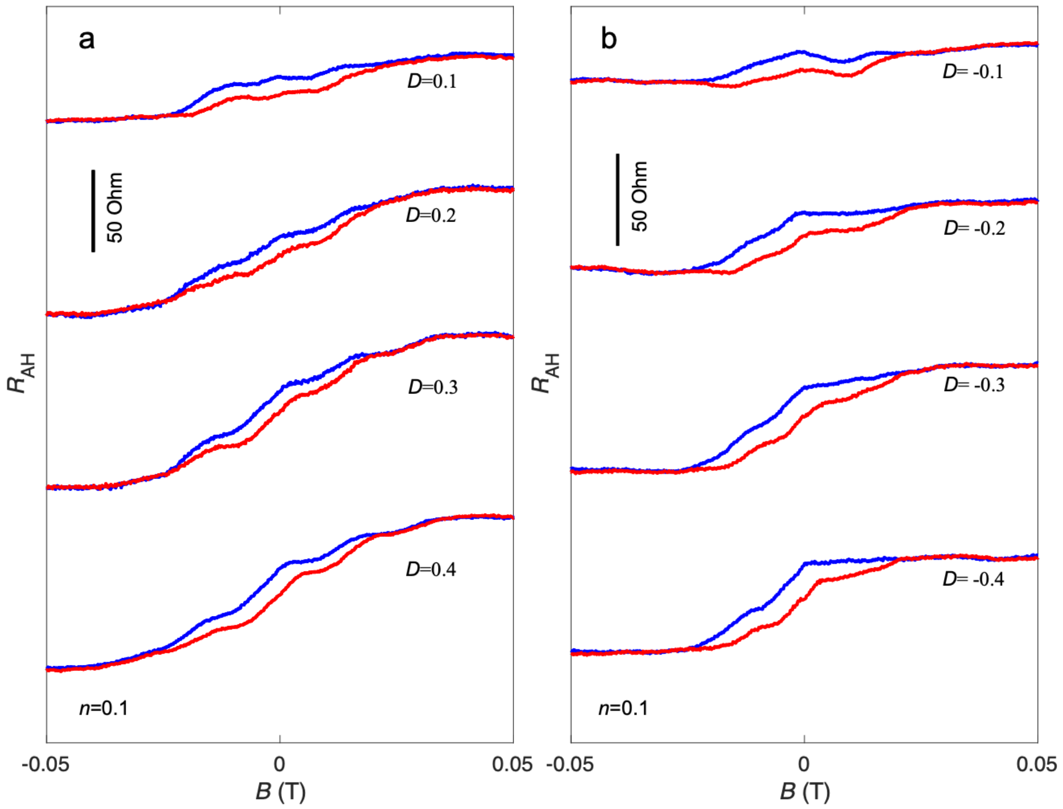

Supplementary Figure 5. *D*-field dependence of anomalous Hall effect. a, Extracted anomalous Hall resistance (R_{AH}) as a function of B at a fixed n=0.1×10^{12} cm^{-2} but different positive D-field conditions. **b**, Same as **a** but for negative D-field conditions. Each pair of curves are shifted vertically for better view. Scale bar is 50 Ohm.

5. AHE on the hole side.

Gate tunable anomalous Hall effect is also observed for the hole side, as shown in Supplementary Fig. 6. Similar to electron side, the hysteretic AHE curve in the hole side becomes more pronounced at lower hole density (see Supplementary Fig. 6a to Supplementary Fig. 6d); AHE seems to vanish for hole densities beyond 10^{12} cm^{-2} holes for the hole side, consistent with what is observed for electron side. While the hole side does exhibit some knee-like step features (Supplementary Fig. 6a), they are less pronounced than what we observed for the electron side (Fig. 3 of main

text). Generally the AHE in the hole side reproduced most features as that in electron side.

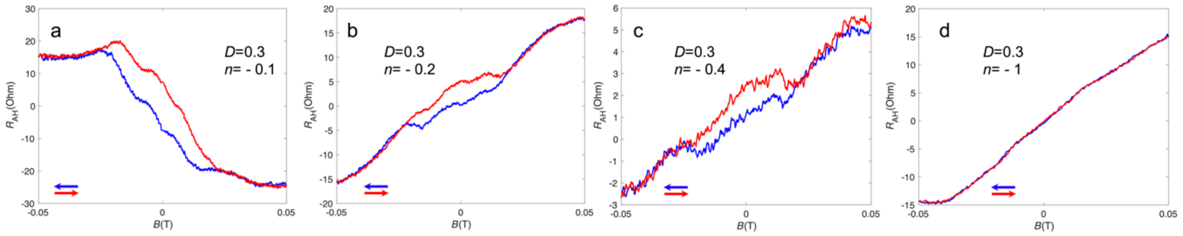

Supplementary Figure 6. Hysteresis and anomalous Hall curves of hole side. **a, b, c&d**, Different carrier density conditions are indicated in the panels at fixed D=0.3 V nm^{-1}. n and D are in units of 10^{12} cm^{-2} and V nm^{-1}, respectively. Here the negative n values indicate hole density.

6. Hall effect at very low density

In this section, we investigate the Hall effect in our devices at very low density. Here we display our Hall effect data for lower densities specifically: n = 0.08, 0.03 and 0.01; these densities are normalized to 10^{12} cm^{-2} plotted as Supplementary Fig. 7 below. We found that AHE persists for densities below n=0.1 (namely n=0.08 and n=0.03, see Supplementary Fig. 7 panel a and b). However, when we pushed to even lower densities (namely n=0.01, see Supplementary Fig. 7 panel c and d), the Hall effect becomes large and does not have the same linear in B background (at higher B fields) as before; it deviates significantly from the form $R_{xy} = \eta B + R_{AH}$ that the Hall effect at higher densities seem to follow which makes it difficult for R_{AH} to be disentangled from the Hall signal. Instead, large hysteretic curves in R_{xy} with a complex form can be observed (see Supplementary Fig. 7 panel c and d). These may arise from a number of sources, e.g., from magnetic domains, from electron-hole puddles that become polarized (valley polarized and/or momentum polarized), disorder, or a combination of all the above. Indeed, we note that even in high mobility graphene heterostructures, electron-hole puddles are of the order δn ~ several x 10^{10} cm^{-2} see e.g., Ref [2,3].

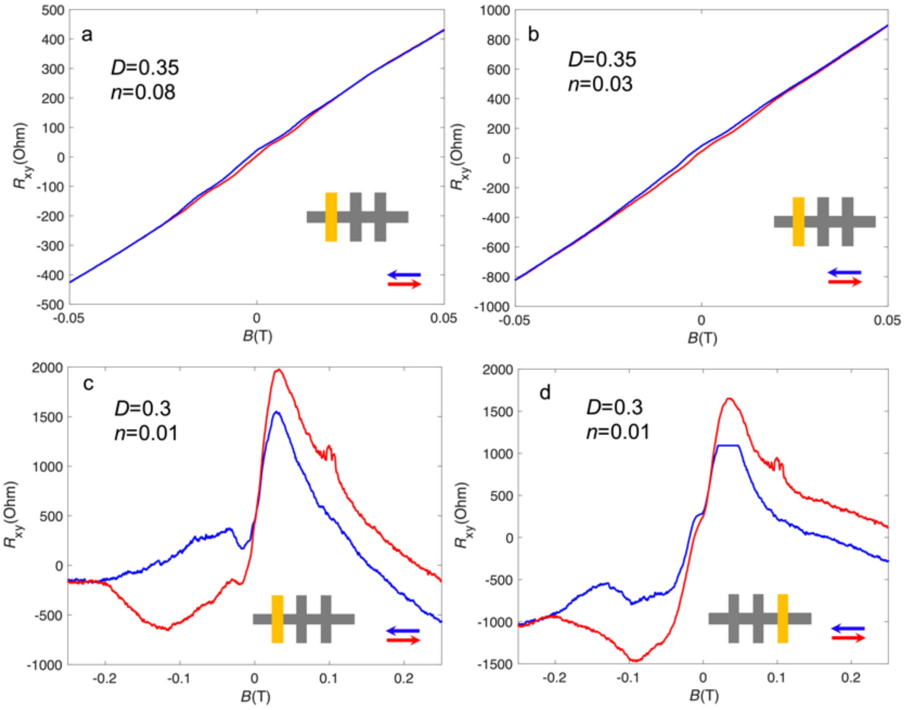

Supplementary Figure 7. a&b, Anomalous Hall (R_{AH}) curves at two very low carrier densities (n indicated in the panels). **c&d**, Hall (R_{xy}) curves at extremely low densities (n indicated in the panels) for two different Hall contacts as denoted in inset. n and D are in units of 10^{12} cm^{-2} and V nm^{-1}, respectively.

7. Extracted ΔR_{AH} as function of n and D conditions

As an illustration of the (n, D) dependence of AHE effect, we extract edthe ΔR_{AH} values for the difference between backward and forward sweep curves of R_{xy} in a B-field scan and plotted these as function of (n, D) states in Supplementary Fig. 8. As shown in the colormap, we can see that regions with finite ΔR_{AH} values are consistent with the region of VM states in Fig. 2 in the main text.

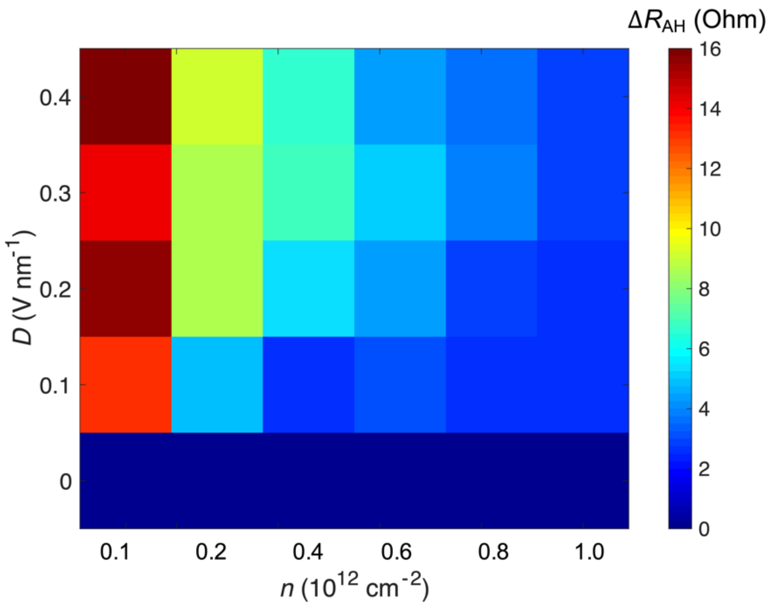

Supplementary Figure 8. ΔR_{AH} as function of n and D conditions at B=0T at 0.3 K.

8. Hamiltonian for tetralayer graphene

We model the Bernal stacked tetralayer graphene using an eight band tight binding model around the Dirac points which can be expressed in terms of two coupled blocks of Bernal stacked bilayer graphene[4,5]. In the basis of $(A_1, B_1, A_2, B_2, A_3, B_3, A_4, B_4)$ the Hamiltonian can be written as

$$\mathcal{H} = \begin{pmatrix} H_{AB}^{12} & W \\ W^{\dagger} & H_{AB}^{34} \end{pmatrix} \quad (1)$$

Here H_{AB}^{12} and H_{AB}^{34} are the Hamiltonian blocks for bilayer graphene

$$H_{AB}^{12} = H_{AB}^{34} = \begin{pmatrix} 0 & v_0\pi^{\dagger} & -v_4\pi^{\dagger} & v_3\pi \\ v_0\pi & \delta & \gamma_1 & -v_4\pi^{\dagger} \\ -v_4\pi & \gamma_1 & \delta & v_0\pi^{\dagger} \\ v_3\pi^{\dagger} & -v_4\pi & v_0\pi & 0 \end{pmatrix} \quad (2)$$

which are coupled by

$$W = \begin{pmatrix} \frac{\gamma_2}{2} & 0 & 0 & 0 \\ 0 & \frac{\gamma_5}{2} & 0 & 0 \\ -\tilde{v}_4\pi & \Gamma_1 & \frac{\gamma_5}{2} & 0 \\ \tilde{v}_3\pi^\dagger & -\tilde{v}_4\pi & 0 & \frac{\gamma_2}{2} \end{pmatrix} \tag{3}$$

Here $\pi = \zeta k_x + ik_y$ with $\zeta = \pm$ being the valley index. Parameters $v_i = \sqrt{3}a_0\gamma_i/2$ ($\tilde{v}_i = \sqrt{3}a_0\Gamma_i/2$) are obtained in terms of tight binding parameters γ_i (Γ_i), and $a_0 = 2.46$ Å is the lattice constant for monolayer graphene. Additionally, δ denotes the potential energy difference between dimer (B_1, A_2, B_3, A_4) and non-dimer (A_1, B_2, A_3, B_4) sites[5]. Without loss of generality, we have set the on-site energy to be zero[1,4]. The values of tight binding parameters[5,6] along with their values are indicated in Supplementary Fig. 9a.

Next, we consider the effect of an out-of-plane displacement field. Here we adopt a "toy model" to mimic the layer screening effects that can occur in multilayer graphene described in e.g., Ref. 7. In particular, we use an unequal potential drop so that the displacement field only affects the outermost layers and middle layers remain neutral. This modifies the upper and bottom bilayer graphene blocks as

$$H_{AB}^{12} \to H_{AB}^{12} + \begin{pmatrix} \Delta & 0 & 0 & 0 \\ 0 & \Delta & 0 & 0 \\ 0 & 0 & 0 & 0 \\ 0 & 0 & 0 & 0 \end{pmatrix}$$
$$H_{AB}^{34} \to H_{AB}^{34} + \begin{pmatrix} 0 & 0 & 0 & 0 \\ 0 & 0 & 0 & 0 \\ 0 & 0 & -\Delta & 0 \\ 0 & 0 & 0 & -\Delta \end{pmatrix} \tag{4}$$

where we have defined $\Delta = -e(V_t - V_b)/2$ for $e > 0$ with V_t and V_b being the voltage at top and bottom gates respectively. For a model, where an equal potential drop is sustained across all layers (including middle layers) is used, see Supplementary Section 9.

In the absence of displacement field, i.e., at $\Delta = 0$ tetralayer graphene is semimetallic, and insulating phase with tunable Fermi surface topology can be enabled at $\Delta \neq 0$. This is illustrated in Supplementary Fig. 9. It is noted that due to screening in graphene multilayer systems theoretical value of Δ can differ from the one imposed by the displacement field *D* [5].

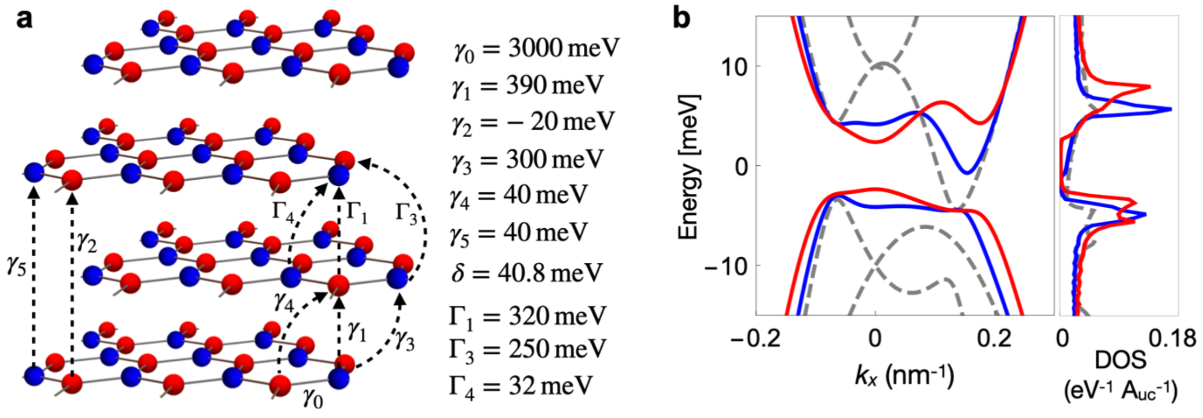

Supplementary Figure 9. a, Crystal structure of Bernal stacked tetralayer graphene along with tight binding parameters used in the calculation. Here, red and blue balls A and B lattice sites respectively. Hopping strengths γ_i and Γ_i are indicated in the crystal structure, and δ is the potential energy difference between dimer and non-dimer sites. **b**, Displacement field tunable band structure and density of states of electrons around Dirac point in Bernal stacked tetralayer graphene using the model described in Supplementary Section 6. Here, we show $\Delta = 0$ (dashed grey line), $\Delta = 20$ meV (blue line) and $\Delta = 40$ meV (red line) for illustration of transition from semimetal at zero displacement field to gapped phase at sufficiently large displacement field. A_{uc} denotes the area of the unit cell.

7. Calculation of Berry curvature and Berry Flux

As described above, a finite displacement field enables a gapped band structure in tetralayer graphene. The displacement field breaks inversion symmetry and produces a Berry curvature distribution in the valleys of BTG; as discussed in the main text a finite Berry flux can manifest as an anomalous Hall effect. For a given Bloch band α with eigenstate $|u_\alpha(\boldsymbol{k})\rangle$, the Berry curvature is given as $\Omega^{\alpha}_{ab}(\boldsymbol{k}) =$

$\partial_{k_a} A_b^\alpha(\boldsymbol{k}) - \partial_{k_b} A_a^\alpha(\boldsymbol{k})$ where $\boldsymbol{A}^\alpha = \langle u_\alpha(\boldsymbol{k})|i\nabla_{\boldsymbol{k}} u_\alpha(\boldsymbol{k})\rangle$ is the Berry connection; Greek and Latin indices denote band and direction respectively. However, the given form of Berry curvature is problematic for numerical evaluations due to the gauge dependency of the Berry connection. Therefore, for numerical convenience we follow the standard approach in Ref. 8 to calculate the Berry curvature using the method of taking small gauge invariant loops (Wilson Loop) in a discretized Brillouin zone[8]; this ensures gauge invariance and enables fast numerical convergence. Here, the Berry curvature can be obtained as a gauge invariant Wilson loop

$$\Omega_{ab}^\alpha(\boldsymbol{k}) = -\frac{i}{\delta k_a \delta k_b} \ln\left[\frac{U_a^\alpha(\boldsymbol{k}) U_b^\alpha(\boldsymbol{k}+\delta k_a)}{U_a^\alpha(\boldsymbol{k}+\delta k_b) U_b^\alpha(\boldsymbol{k})}\right] \tag{5}$$

around a given $\boldsymbol{k}$-point formed by links $U_a^\alpha(\boldsymbol{k}) = \frac{\langle u_\alpha(\boldsymbol{k})|u_\alpha(\boldsymbol{k}+\delta k_a)\rangle}{|\langle u_\alpha(\boldsymbol{k})|u_\alpha(\boldsymbol{k}+\delta k_a)\rangle|}$ with $\delta k_a \times \delta k_b$ describing the mesh size of the discrete Brillouin zone. Using the Berry curvature obtained from Eq. (5) we evaluate the Berry flux

$$F_{ab} = \sum_{\alpha,\boldsymbol{k}} f_\alpha(\boldsymbol{k}) \Omega_{ab}^\alpha(\boldsymbol{k}) \tag{6}$$

Where $f_\alpha(\boldsymbol{k})$ is the Fermi function and the sum of band indices runs over all filled bands. The calculation is done on a grid of 400×400 $\boldsymbol{k}$-points around the Dirac point in each valley. Berry curvature for conduction band in K-valley for $\Delta = 20$ meV and $\Delta = 40$ meV is shown in Supplementary Fig. 10.

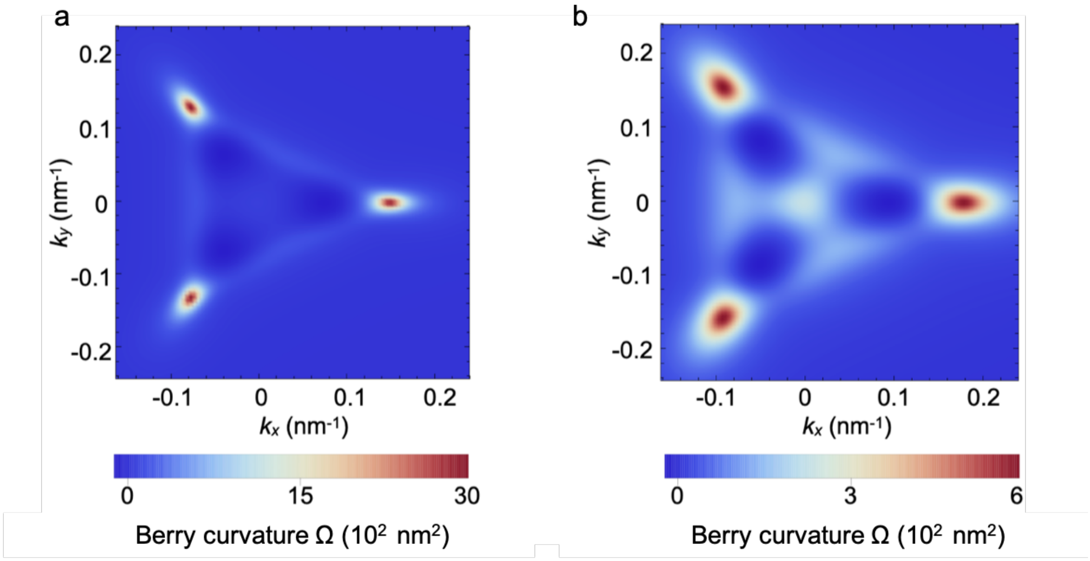

Supplementary Figure 10. Gate tunable Berry curvature in BTG. Here for illustration we show $\Delta = 20$ meV (**a**) and $\Delta = 40$ meV (**b**).

8. Calculation of orbital magnetic moment

We obtain the magnetic moment in BTG using the multiband formula[9], so that for a given Bloch band α

$$m_\alpha(\boldsymbol{k}) = \frac{e}{\hbar} Im \sum_{\beta \neq \alpha} \frac{\langle u_\beta(\boldsymbol{k}) | \partial_{k_x} H(\boldsymbol{k}) | u_\alpha(\boldsymbol{k}) \rangle \left\langle u_\alpha(\boldsymbol{k}) \left| \partial_{k_y} H(\boldsymbol{k}) \right| u_\beta(\boldsymbol{k}) \right\rangle}{\epsilon_\beta(\boldsymbol{k}) - \epsilon_\alpha(\boldsymbol{k})} \quad (7)$$

where $\epsilon_\alpha(\boldsymbol{k})$ is the energy. In evaluating the magnetic moment in last equation we consider all the eight bands of Hamiltonian in Eq. (1). Similar to Berry curvature, orbital magnetic moment also evolves with the displacement field as shown in Supplementary Fig. 11 for conduction band in K-valley.

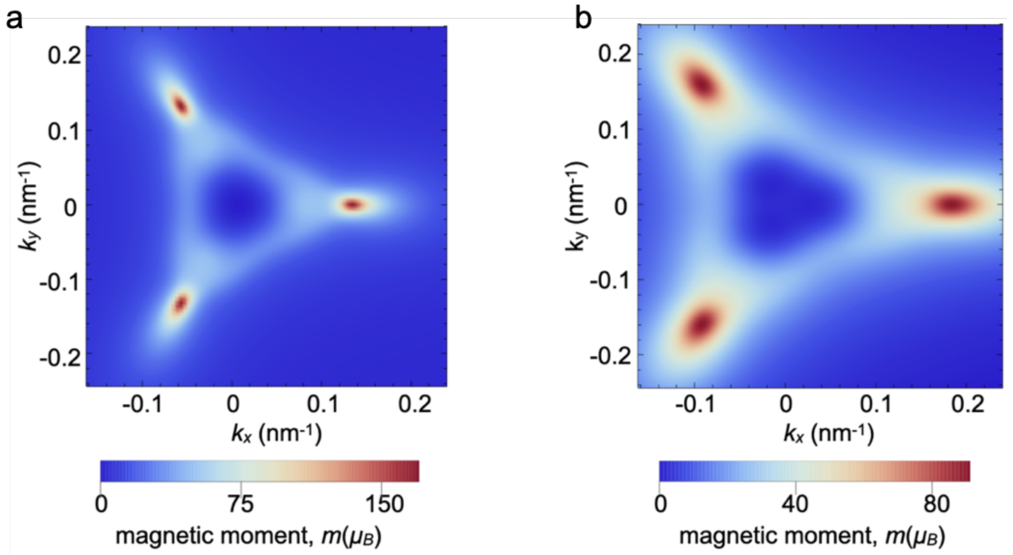

Supplementary Figure 11. Gate tunable orbital magnetic moment in BTG. Here for illustration we show $\Delta = 20$ meV (**a**) and $\Delta = 40$ meV (**b**).

9. Band structure and Berry curvature with finite potential on middle layers

In the main text and SI sections 6,7 and 8 we have considered an unequal potential drop "toy model" that screens the electric field across the middle layers. Here, we consider the case when the electric field is not completely screened. We consider the electric field to act on four layers such that the corresponding potential drop across the layers is (Δ, Δ/3, -Δ/3, -Δ)[10]. Here we have maintained a total difference 2Δ between outermost layers for ease of comparison with the unequal potential drop "toy model" described in Equation S4. Below using the equal potential drop model, we plot the band structure for Δ = 0, 20, 40 meV and Berry curvature for Δ = 40 meV in Supplementary Fig. 12. As can be seen in Supplementary Figure 12, while having a finite potential drop across middle layers changes the band structure and Berry curvature distribution quantitatively, it does not affect our qualitative conclusions.

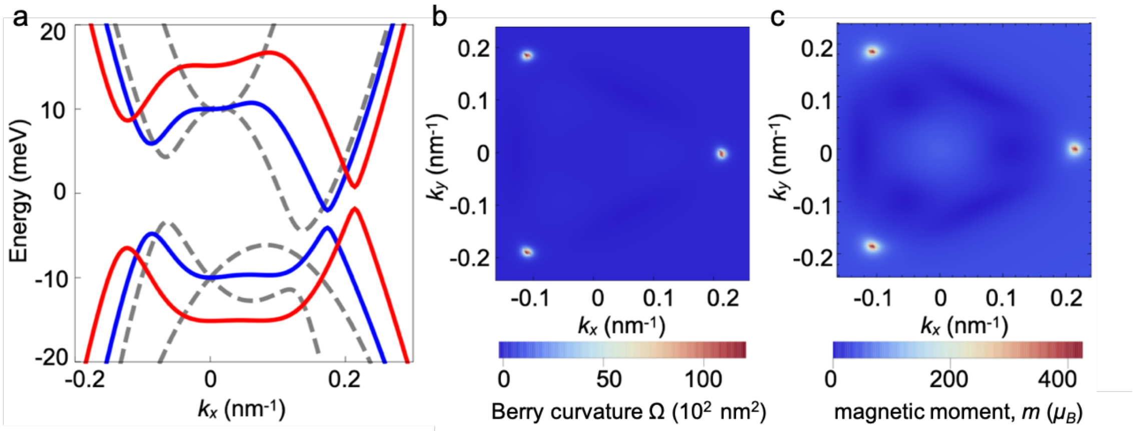

Supplementary Figure 12. (**a**) Band structure (for Δ = 0 [dashed], 20 [blue], 40 [red] meV) and (**b**) Berry curvature (for Δ = 40 meV) and (**c**) magnetic moment (for Δ = 40 meV) in Bernal stacked tetralayer graphene using an equal potential drop across all the layers, see text in Supplementary Section 9 for the model.

10. Reproducibility of staircase feature in AHE.

The staircase-like AHE in BTG (Fig. 3 in main text) of the main text is an intriguing feature. To investigate the reproducibility of this feature, multiple measurements at the same (n, D) condition are carried out. As shown in Supplementary Fig. 13, with applied current of 50 nA and 100 nA (for three times), the staircase features are all well resolved.

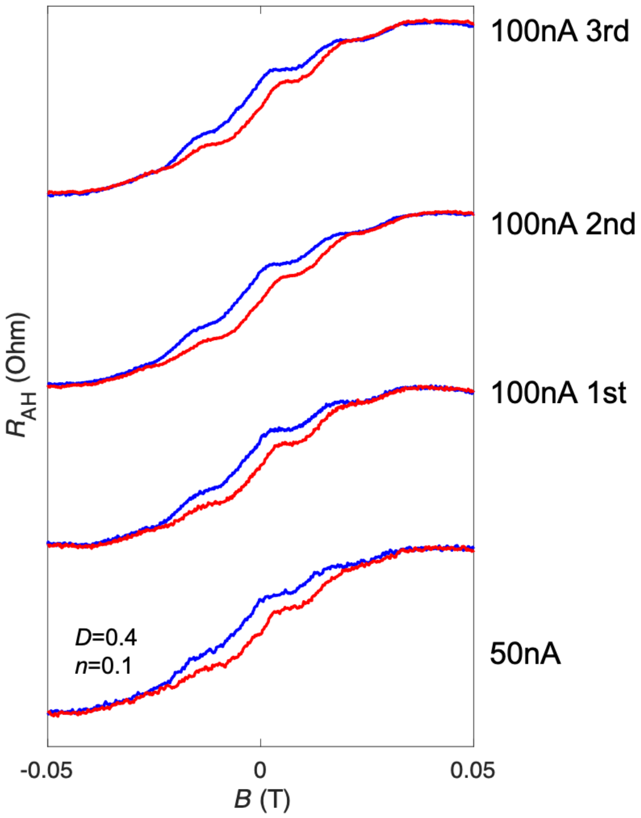

Supplementary Figure 13. Reproducibility of staircase-like AHE curves. From bottom to top is the measurement results at 50nA condition and 100nA condition for successive three times. *n* and *D* are in units of 10^{12} cm^{-2} and V nm^{-1}, respectively.

11. AHE in additional devices.

In addition to the device shown in main text, we have also fabricated three more devices, where AHE are all observed and shown in Supplementary Fig. 14. Specific (*n*, *D*) conditions are denoted in corresponding panels, which substantiate the AHE as an controllable and reproducible effect in BTG.

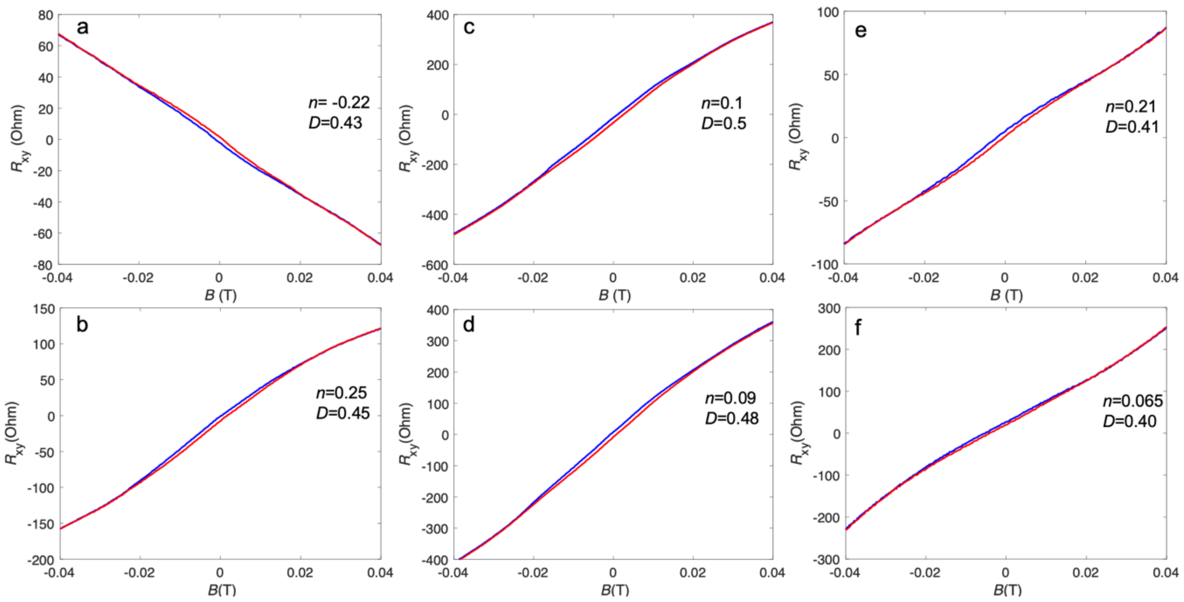

Supplementary Figure 14. AHE results from additional samples. a,b,c&d, Hall resistance (raw data) results obtained in device BTG_2 at different (n,D) conditions. **e&f,** Hall resistance (raw data) obtained in device BTG_3 and BTG_4, respectively. *n* and *D* are in units of 10^{12} cm^{-2} and V nm^{-1}, respectively.

References

1. Zhou, H. *et al*. Half and quarter metals in rhombohedral trilayer graphene, *Nature* **598**, 429-433 (2021).

2. Martin, J. *et al*. "Observation of electron–hole puddles in graphene using a scanning single-electron transistor." *Nature physics* 4.2 (2008): 144-148.

3. Deshpande, A. *et al*. "Spatially resolved spectroscopy of monolayer graphene on SiO 2." Physical Review B 79.20 (2009): 205411.

4. Koshino, M. & McCann, E. Landau level and the quantum Hall effect of multilayer graphene. *Phys. Rev. B* **83**, 165443 (2011).

5. Shi, Y. *et al.* Tunable Lifshitz transitions and multiband transport in tetralayer Graphene. *Phys. Rev. Lett.* **120**, 096802 (2018).

6. Che, S. *et. al.* Helical edge states and quantum phase transitions in tetralayer graphene. *Phys. Rev. Lett.* **125**, 036803 (2020).

7. Koshino, M., Interlayer screening effect in graphene multilayers with ABA and ABC stacking, *Phys Rev B* 81, 125304 (2010).

8. Fukui, T., Hatsugai, Y. & Suzuki, H. Chern numbers in discretized Brillouin zone: Efficient method of computing (spin) Hall conductances. *J. Phys. Soc. Japan* **74**, 1674-1677 (2005).

9. Xiao, D., Chang, M.-C. & Niu, Q. Berry phase effects on electronic properties. *Rev. Mod. Phys.* **82**, 1959-2007 (2010).

10. Ghazaryan, A., Holder, T., Berg, E. & Serbyn M. Multilayer graphenes as a platform for interaction-driven physics and tological superconductivity. *Phys. Rev. B* **107**, 104502 (2023).
